# Diagnostic Agreement for High-Grade Urothelial Cell Carcinoma in Atypical Urine Cytology: A Nationwide Survey Reveals a Tendency for Overestimation in Specimens with an N/C Ratio Approaching 0.5

**DOI:** 10.3390/cancers12020272

**Published:** 2020-01-22

**Authors:** Yeh-Han Wang, Jen-Fan Hang, Chien-Hui Wen, Kuan-Cho Liao, Wen-Ying Lee, Chiung-Ru Lai

**Affiliations:** 1 Department of Anatomic Pathology, Taipei Institute of Pathology, Taipei 10374, Taiwan; yehanwang@gmail.com; 2 Institute of Public Health, National Yang-Ming University, Taipei 11221, Taiwan; 3 College of Nursing, National Taipei University of Nursing and Health Sciences, Taipei 11219, Taiwan; 4 Department of Pathology and Laboratory Medicine, Taipei Veterans General Hospital, Taipei 11217, Taiwan; crlai@vghtpe.gov.tw; 5 School of Medicine, National Yang-Ming University, Taipei 11221, Taiwan; 6 Department of Pathology, Kaohsiung Medical University Hospital, Kaohsiung Medical University, Kaohsiung 80756, Taiwan; daynawen@gmail.com; 7 Department of Radiation Oncology, Kaohsiung Chang Gung Memorial Hospital, Kaohsiung 83301, Taiwan; piko0104@gmail.com; 8 Department of Public Health, College of Health Sciences, Kaohsiung Medical University, Kaohsiung 80708, Taiwan; 9 Department of Cytopathology, Chi Mei Medical Center, Tainan 71004, Taiwan; d940232@mail.chimei.org.tw

**Keywords:** atypical urine cytology, the Paris system (TPS), interobserver concordance, urothelial carcinoma, nuclear-to-cytoplasmic (N/C) ratio

## Abstract

In the Paris System (TPS), standardized cytomorphological criteria and diagnostic categories were proposed for reporting urine cytology. To evaluate the diagnostic agreement and interobserver concordance for assessing TPS criteria, the Taiwan Society of Clinical Cytology organized an online survey with 10 atypical urine cytology cases. A total of 137 participants completed the survey. The mean agreement of diagnosis was 51.2%, ranging from 34.3% to 83.2% for each case. For 60% (6/10) of cases, the agreement was <50%. The interobserver concordance of diagnosis and cytological criteria assessment showed poor agreement. The nuclear-to-cytoplasmic (N/C) ratio had the highest kappa value of 0.386, indicating a significantly higher interobserver concordance and reproducibility than the other three TPS criteria. The correct rate of assessing the N/C ratio increased as the N/C ratio increased (correlation coefficient: 0.891, *p* < 0.01). Three cases with an N/C ratio near 0.5 were overestimated. Poor interobserver concordance of diagnosis and TPS criteria was revealed. Compared with other cytological features, the N/C ratio assessment was quantitative and more reproducible, but a tendency to overestimate cells was noted when the N/C ratio was approximately 0.5. Continuing education programs should emphasize the accurate assessment of N/C ratio to improve the application of TPS.

## 1. Introduction

In the Paris System (TPS) for reporting urine cytology, standardized diagnostic criteria and terminology are proposed. Using four qualitative or semiquantitative criteria of cytomorphological features, such as nuclear-to-cytoplasmic (N/C) ratio, degree of hyperchromasia, nuclear membrane irregularity, and clumping chromatin pattern, the risk of high-grade urothelial carcinoma (HGUC) is stratified into four diagnostic categories: negative for HGUC (NHGUC), atypical urothelial cells (AUC), suspicious for HGUC (SHGUC), and HGUC [1]. After the release of TPS in 2015, the Taiwan Society of Clinical Cytology (TSCC) has arranged several educational programs for introducing TPS and its standardized criteria in 2016 and 2017. However, TPS has not been widely used in Taiwan due to concerns regarding diagnostic variability for indeterminate categories and consequential confusion for clinicians.

Urine cytology is a key diagnostic tool either in screening or follow-up for urothelial malignancy, therefore the consistency and accuracy of cytomorphological assessments are crucial for patient management. The implementation of TPS is mostly considered to improve the risk stratification and diagnostic accuracy of the indeterminate group, decreasing unnecessary AUC diagnosis [2,3,4,5,6,7]. However, a considerable increase in atypical diagnoses has been also reported [8]. The major concern is that some of the criteria are subjective, and thus, assessments may be inconsistent among observers. Long et al. and Kurtycz et al. have noted that the agreement of diagnosis using TPS is not satisfying, especially for indeterminate cases, and the interobserver concordance of diagnosis is low [9,10]. However, they did not further clarify the interobserver concordance of the assessment of cytological features. Furthermore, the relationship between cytological assessments and diagnoses has not been addressed.

To further evaluate the interobserver concordance for assessing atypical urothelial cells and how the cytologists incorporate TPS criteria in real-world practice, the Education and Research Committee of the TSCC designed an online survey. The survey results are presented in this study.

## 2. Results

A total of 137 cytologists (91 cytotechnologists and 46 cytopathologists) responded to the online survey. A summary of the general profile of participants is presented in Table 1.

### 2.1. Overall Agreement

The mean overall agreement of diagnosis was 51.2%. The diagnostic agreement for each case ranged from 34.3% to 83.2% (median: 44.9%). Six of 10 cases had a diagnostic agreement of <50%. Similar results were noted in the assessment of TPS criteria. The overall agreement of the N/C ratio, hyperchromasia, nuclear membrane irregularity, and chromatin clumping was 52.6–97.8% (mean: 65.9%), 46.7–72.3% (mean: 58.1%), 46.7–73% (mean: 60.2%), and 62–97.1% (mean: 79.2%), respectively. The details of each case are provided in Table 2. No significant differences in the assessment and diagnosis were according to different professions, years of practice, and types of practice.

### 2.2. Interobserver Concordance

The interobserver concordance of assessment and diagnosis was demonstrated using Fleiss’ kappa coefficient. Fleiss’ kappa coefficients of N/C ratio, hyperchromasia, nuclear membrane irregularity, and chromatin clumping were 0.386 (95% confidence interval [CI]: 0.381–0.391), 0.128 (95% CI: 0.123–0.133), 0.152 (95% CI: 0.148–0.157), and 0.239 (95% CI: 0.233–0.246), respectively. The kappa of diagnoses was 0.182 (95% CI: 0.178–0.186).

### 2.3. Accuracy of N/C Ratio Assessment

The correct rate of N/C ratio assessments positively correlated with the N/C ratio (correlation coefficient: 0.891, *p* < 0.01). All the cases with N/C ratios >0.7 (range, 0.79–0.86) indicated a high correct rate (>70%). Three cases were overestimated with a <50% correct rate. Two cases with N/C ratios of 0.39 and 0.42 were upgraded to the 0.5–0.7 group and one case with an N/C ratio of 0.58 was upgraded to the >0.7 group (Table 3).

## 3. Discussion

TPS, which emphasizes the standardization of cytological criteria and diagnostic terminology, is thought to improve the agreement and reproducibility of urine cytological diagnosis. The use of TPS has resulted in an increase in specificity and positive predictive values as well as high diagnostic concordance of benign and malignant categories [10,11]. However, it is still difficult to achieve a consistent diagnosis of the atypical indeterminate group between observers with these clear and comprehensive cytological criteria. The agreement of the AUC group was reported to be low and the correct interpretation rate of AUC from using TPS criteria was only 36% in the Paris Interobserver Reproducibility study [9,10]. Our study obtained similar results. Six of the 10 cases had unsatisfying overall diagnostic agreement (<50%). Fleiss’ kappa coefficients further indicated poor interobserver concordance (Fleiss’ kappa = 0.182). Although the agreement of cytology diagnosis seemed to be related to experience in practice, the subgroup analysis of professions, experience, and types of practice revealed no significant differences in overall agreement.

The diagnostic variability in the indeterminate category could be attributed to the inconsistency in assessing the cytological features among observers. Several studies have investigated the performance and use of cytomorphological features for diagnosing HGUC. All four TPS criteria were reported to be crucial indicators in urine cytology diagnosis although the sensitivity, specificity, and risk of malignancy varied in different studies or clinical scenarios [12,13,14,15]. None of the four cytological features can be regarded as a single conclusive criterion for diagnosing HGUC. In addition, the nuclear features of urothelial cells are influenced by specimen degeneration. Urothelial cells are more hyperchromatic with increased nuclear membrane irregularity as they are more degenerated. A lower increase in the N/C ratio was noted as the nucleus could be more condensed [2]. The assessment of cytomorphological features can be less consistent and subjective as per the various degrees of cell degeneration in each specimen. In terms of qualitative criteria, such as degrees of hyperchromasia or nuclear membrane irregularity, no definitive cutoffs could be applied. Glass et al. noted poor interobserver concordance for assessing cytological features and considered that the features of atypical cells are less objective than those of definite malignant cells [16].

In our survey, the overall agreement of assessments using TPS criteria was similar to that of diagnosis. The interobserver concordance was unacceptable (kappa < 0.4). However, among the four TPS criteria, the N/C ratio assessment revealed the highest kappa value (0.386), which was close to the cutoff of 0.4, indicating a fair to good agreement; this value was significantly higher than the kappa values for the other three TPS criteria. This indicated that the N/C ratio assessment was more reproducible between observers than evaluations of hyperchromasia, irregular nuclear membrane, and clumping chromatin when assessing atypical urothelial cells.

Furthermore, compared with the other three TPS criteria, N/C ratio was found to be the only objective and quantitative indicator for stratifying urothelial cells in different diagnostic categories. Given that the N/C ratio of 0.5 was proven to be a rational cutoff value for defining urothelial cells as being atypical [17], the accuracy and reproducibility in visual assessment were still not perfect. Zhang et al. reported a tendency of overestimation in morphological assessments of N/C ratio, especially when it is close to the cutoff value [18]. Layfield et al. indicated that the assessment of a 0.5–0.7 N/C ratio may be insufficiently accurate for diagnosis [19]. However, the N/C ratio is considered the most restrictive criterion for diagnosing HGUC [2].

According to TPS, accurately assessing the N/C ratio is crucial. In this survey, among the six cases demonstrating <50% overall agreement in diagnosis, half of them (3/6) had a borderline (0.5–0.7) N/C ratio. We also found that cases with an N/C ratio of approximately 0.5 were likely to be overestimated. Thirty percent of the cases (3/10; 2 cases: <0.5 N/C ratio; 1 case: 0.5–0.7 N/C ratio) were overestimated and upgraded by most participants. However, a higher correct rate was observed for the representative cells with a >0.7 N/C ratio and the overall agreement positively correlated with the N/C ratio for atypical urothelial cells. Overestimation of the borderline N/C ratio and a high correct rate for assessing an N/C ratio of >0.7 were both noted in our study, reflecting fair interobserver concordance and reproducibility, as demonstrated by Fleiss’ kappa coefficient. Therefore, an accurate assessment of the N/C ratio cutoff is essential as the overestimation probably leads to an overcall in benign cases or would upgrade AUC cases to the SHGUC or HGUC category. In the aforementioned two scenarios, the 0.5 cutoff should be further emphasized in practice because overestimating this cutoff would overcall benign or reactive urothelial cells as being in the AUC category, resulting in unnecessary follow-up. In addition, the high N/C ratio (>0.7) can be the most reproducible parameter for detecting HGUC in atypical urine cytology.

Several limitations could have influenced the results of this survey. First, only two photographs were provided for cytological diagnosis. Compared with scenarios in daily practice, the cytological information available was limited. Participants might have faced difficulty in diagnosis with such few dispersed or clustered urothelial cells as well as limited background information. In addition, applying the principle of cell numbers is essential for differentiating cases of SHGUC from those of HGUC. Following the TPS criteria strictly would prevent participants from diagnosing HGUC in this setting, and thus, would decrease the agreement of diagnosing SHGUC or HGUC in the survey.

Second, the kappa value correlates with the number of categories and observers. Up to 137 participants joined the survey and only 10 cases were included for providing answers. This possibly decreased the kappa value in the statistical analysis. Owing to the limitation of the small case number in this survey, performing further subgroup analyses with Fleiss’ kappa coefficients was not possible. However, participants may have been more distracted if too many cases were included in one questionnaire, especially as Google Forms does not provide the option of temporarily saving answers. The intraobserver consistency and accuracy might be lowered in such a case. This 10-case study design aimed to achieve a balance of analytic power and data correctness.

Although some publications have provided the reference cutoffs of kappa values for stratifying and categorizing agreement [20,21,22], it could be misleading. With fewer categories it is easier to obtain a higher kappa value [23]. Therefore, the kappa values of clumping chromatin (two-tier options) and diagnosis (four-tier options) were not comparable with the other three criteria, which could have led to an overestimation and underestimation of interobserver concordance, respectively.

Finally, in this study, only four major TPS cytomorphological criteria were included. Other informative and indicative cytological features, such as anisonucleosis, large nucleoli, cell size, and isolated or clustered atypical cells, were not analyzed. Such parameters could influence observers during practice or diagnosis. Thus, without the further collection and analysis of these parameters in this survey, it could have biased the agreement and variability of cytological diagnoses in this study.

## 4. Materials and Methods

We designed an online survey consisting of 10 urine cytology cases. The survey was conducted using Google Forms, which is a free online survey application. This application allows users to design and establish personalized online questionnaires. Uploading photographs is possible, allowing designers to present their surveys with both text and illustration. The survey had been open for 6 weeks from June 2017. In all 10 urine cytology cases in this survey, various degrees of cytological atypia were revealed. HGUC was confirmed in eight cases based on the surgical specimens obtained either from biopsy or transurethral resection. In the remaining two cases, no evidence of malignancy was present during follow-up.

**Figure 1:**
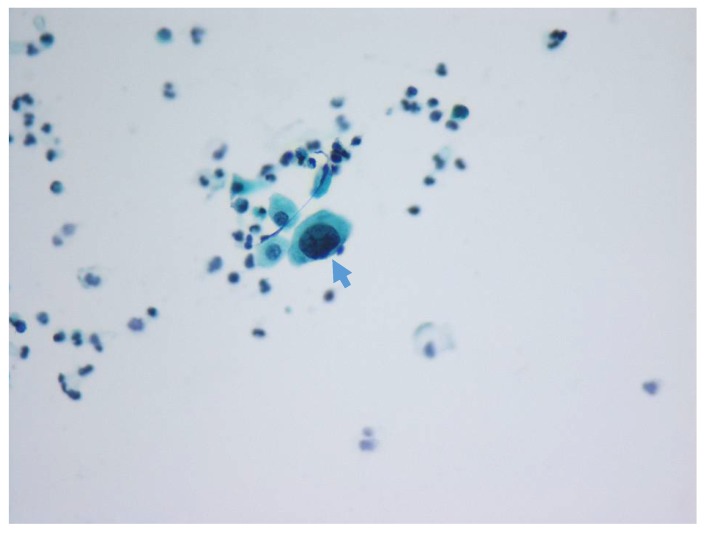
100× image provided in the survey, which includes the target cell and adjacent cells with more background information for participants (arrow: representative cell, which would be demonstrated in another 400× image separately).

In the online questionnaire, the participants were requested to provide categorical information of their professions (e.g., whether they were cytotechnologists or cytopathologists), years of practice (<5, 5–10, 10–20, and >20 years), and types of practice (e.g., medical center, regional/local hospital, and commercial lab). Assessment of each case started with a high-power (400×) image of one representative urothelial cell. The participants had to provide answers for each image based on the N/C ratio, degree of hyperchromasia, presence of nuclear membrane irregularity, and presence of chromatin clumping. In the following questions, a medium-power (100×) image (Figure 1) was presented to provide more morphological information regarding the adjacent urothelial cells (either in small clusters or a single cell in the background). The participants were then requested to select one diagnosis out of the four TPS categories (Table 4).

**Figure 2:**
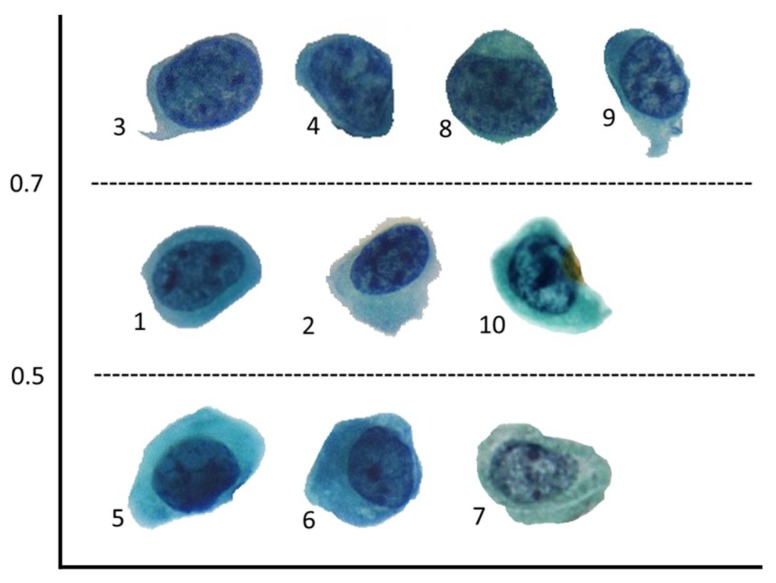
N/C ratio ranges of the representative urothelial cells in the online urine cytology survey. The small numbers over the lower left side near the cells indicate the question number in the online survey.

To avoid observer and selection bias, the distribution of the N/C ratio was balanced among three ranges of <0.5, 0.5–0.7, and >0.7. The cutoffs were defined in TPS. The N/C ratio of the representative urothelial cells was calculated using ImageJ software in advance, and the order of the 10 cases was randomly assigned (Figure 2). We analyzed the correct rate and correlation of assessments made by participants based on the exact N/C ratio.

### Statistical Analysis

The summary spreadsheet was automatically generated by Google Forms. Duplicate responses due to repeated submissions from any single participant were counted only once.

In terms of agreement and reproducibility, two types of concordances were presented and discussed in this study. First, overall agreement was analyzed, which provides a general picture of the agreement of diagnosis and cytological assessment in each case. Second, interobserver concordance was investigated, which provides details on the variability and reproducibility between observers.

To demonstrate the overall agreement of diagnosis and assessment of cytological criteria in practice, we used descriptive statistics to reveal the most selected diagnoses and cytological features of each case. A chi-squared test was used to analyze differences in subgroup assessments.

To investigate the interobserver concordance in assessments, Fleiss’ kappa coefficient was calculated. The kappa value ranges from −1 to 1. A larger kappa value represents higher agreement between observers. Fleiss et al. proposed a simplified classification for interpreting kappa values. They used cutoff values of 0.4 and 0.75 to stratify interobserver concordance into three categories of poor (<0.4), fair to good (0.41–0.75), and excellent agreement [22].

## 5. Conclusions

Although TPS is believed to be useful and easily adopted in daily practice in urine cytology diagnosis, this nationwide online survey in Taiwan revealed low interobserver concordance for assessing the cytomorphological features of atypical urothelial cells. Although the overall agreement was higher when the N/C ratio was >0.7, observers tended to overestimate the N/C ratio of cells approached 0.5, which may have led to overcall in classifications. Therefore, accurate assessment of the N/C ratio close to cutoffs is a key training objective and should be emphasized in continuing education or training programs related to TPS. In addition, employment of ancillary tests, such as immunocytochemistry for p16 and Ki-67 or Urovysion fluorescence in situ hybridization (FISH), may help improve the risk stratification in the intermediate group [24,25]. A combination of cytomorphological assessment and ancillary testing would provide more accurate results for patient management.

## Figures and Tables

**Table 1:** Summary of the Participants’ Profiles.

Profession (*n* = 137)	Number
Cytotechnologist	91
Cytopathologist	46
Years of Practice (*n* = 137)	
<5 years	43
5–10 years	32
10–20 years	47
>20 years	15
Types of practice (*n* = 137)	
Medical center	48
Regional/Local hospital	68
Central laboratory	21

**Table 2:** Summary of the most favored answer from participants.

Case	NC Ratio	Hyperchromasia	Nuclear Membrane Irregularity	Clumping Chromatin	Diagnosis
1	>0.7 75 (54.74%)	moderate to severe 69 (50.36%)	mild 100 (72.99%)	yes 105 (76.64%)	AUC/SHGUC * 47 (34.30%)
2	0.5–0.7 94 (68.61%)	moderate to severe 98 (71.53%)	mild 70 (51.09%)	yes 122 (89.05%)	AUC 56 (40.88%)
3	>0.7 134 (97.81%)	Mild 64 (46.72%)	no 95 (69.34%)	yes 85 (62.04%)	SHGUC 57 (41.61%)
4	>0.7 129 (94.16%)	moderate to severe 83 (60.58%)	severe 78 (56.93%)	yes 126 (91.97%)	HGUC 79 (57.66%)
5	0.5–0.7 109 (79.56%)	Mild 70 (51.09%)	mild 91 (66.42%)	yes 98 (71.53%)	AUC 78 (56.93%)
6	0.5–0.7 72 (52.55%)	Mild 81 (59.12%)	mild 91 (66.42%)	no 89 (64.96%)	NHGUC 62 (45.26%)
7	<0.5 81 (59.12%)	No 75 (54.74%)	no 86 (62.77%)	no 95 (69.34%)	NHGUC 114 (83.21%)
8	>0.7 105 (76.64%)	moderate to severe 76 (55.47%)	mild 83 (60.58%)	yes 124 (90.51%)	SHGUC 58 (42.34%)
9	>0.7 103 (75.18%)	moderate to severe 99 (72.26%)	severe 67 (48.91%)	yes 133 (97.08%)	SHGUC 90 (65.69%)
10	0.5–0.7 82 (59.85%)	moderate to severe 82 (59.85%)	mild 64 (46.72%)	yes 108 (8.83%)	AUC 61 (44.53%)

* The two diagnostic categories selected by the same number of the participants. AUC: atypical urothelial cells, HGUC: high-grade urothelial carcinoma, NHGUC: negative for high-grade urothelial carcinoma, SHGUC: suspicious for high-grade urothelial carcinoma.

**Table 3:** The correct rate of nuclear-to-cytoplasmic (N/C) ratio assessment.

Case No.	N/C Ratio	Correct N/C Range (% of Correct Rate)	Most Favored Assessment of N/C Range (% of Response)
3	0.84	>0.7 (97.8%)	As left
4	0.85	>0.7 (94.2%)	As left
8	0.79	>0.7 (76.6%)	As left
9	0.86	>0.7 (75.2%)	As left
2	0.54	0.5–0.7 (68.6%)	As left
10	0.65	0.5–0.7 (59.9%)	As left
7	0.43	<0.5 (59.1%)	As left
6	0.42	<0.5 (46.7%)	0.5–0.7 (52.6%)
1	0.58	0.5–0.7 (43.8%)	>0.7 (54.7%)
5	0.39	<0.5 (14.6%)	0.5–0.7 (79.6%)

**Table 4:** Survey Form (translated) *.

Case No.	
400× photo (including only one target cell)	
Range of N/C ratio?	Irregular nuclear membrane?
□ <0.5	□ No
□ 0.5–0.7	□ Yes, but minimal
□ >0.7	□ Yes, prominent
Degrees of hyperchromasia?	Coarse/Clumping chromatin?
□ None	□ No
□ Mild	□ Yes
□ Moderate to severe	
**100× photo (including several cells in clusters or disperse)**	
Your diagnosis?	
□ Negative for HGUC (NHGUC)	
□ Atypical urothelial cells (AUC)	
□ Suspicious for HGUC (SHGUC)	
□ HGUC	

* The original questionnaire is made in Traditional Chinese. HGUC: high-grade urothelial carcinoma.

## References

[1] Rosenthal D.L., Wojcik E.M., Kurtycz D.F.I. (2015). The Paris System for Reporting Urinary Cytology.

[2] Cowan M.L., Rosenthal D.L., VandenBussche C.J. (2017). Improved risk stratification for patients with high-grade urothelial carcinoma following application of the Paris system for reporting urinary cytology. Cancer.

[3] Hassan M., Solanki S., Kassouf W., Kanber Y., Caglar D., Auger M., Brimo F. (2016). Impact of implementing the paris system for reporting urine cytology in the performance of urine cytology: A correlative study of 124 cases. Am. J. Clin. Pathol..

[4] Northrup V., Acar B.C., Hossain M., Acker M.R., Manuel E., Rahmeh T. (2018). Clinical follow up and the impact of the Paris system in the assessment of patients with atypical urine cytology. Diagn. Cytopathol..

[5] Malviya K., Fernandes G., Naik L., Kothari K., Agnihotri M. (2017). Utility of the Paris system in reporting urine cytology. Acta Cytol..

[6] Torous V.F., Brancely D., VanderLaan P.A. (2017). Implementation of the paris system for reporting urinary cytology results in lower atypical diagnostic rates. J. Am. Soc. Cytopathol..

[7] Meilleroux J., Daniel G., Aziza J., d’Aure D.M., Quintyn-Ranty M.L., Basset C.M., Evrard S.M., Courtade-Saidi M.M. (2018). One year of experience using the Paris system for reporting urinary cytology. Cancer Cytopathol..

[8] Granados R., Duarte J.A., Corrales T., Camarmo E., Bajo P. (2017). Applying the Paris system for reporting urine cytology increases the rate of atypical urothelial cells in benign cases: A need for patient management recommendations. Acta Cytol..

[9] Long T., Layfield L.J., Esebua M., Frazier S.R., Giorgadze D.T., Schmidt R.L. (2017). Interobserver reproducibility of the paris system for reporting urinary cytology. Cytojournal.

[10] Kurtycz D.F.I., Barkan G.A., Pavelec D.M., Rosenthal D.L., Wojcik E.M., VandenBussche C.J., Mangiulli K., Olson M.T. (2018). Paris interobserver reproducibility study (PIRST). J. Am. Soc. Cytopathol..

[11] Stanzione N., Ahmed T., Fung P.C., Cai D., Lu D.Y., Sumida L.C., Moatamed M.D.N. (2020). The continual impact of the Paris system on urine cytology, a three year experience. Cytopathology.

[12] Mokhtar G.A., Al-Dousari M., Al-Ghamedi D. (2010). Diagnostic significance of atypical category in the voided urine samples: A retrospective study in a tertiary care center. Urol. Ann..

[13] Deshpande V., McKee G.T. (2005). Analysis of atypical urine cytology in a tertiary care center. Cancer.

[14] VandenBussche C.J., Sathiyamoorthy S., Owens C.L., Burroughs F.H., Rosenthal D.L., Guan H. (2013). The Johns Hopkins hospital template for urologic cytology samples: Parts ii and iii: Improving the predictability of indeterminate results in urinary cytologic samples: An outcomes and cytomorphologic study. Cancer Cytopathol..

[15] Pierconti F., Martini M., Straccia P., Fiorentino V., Musarra T., Larocca L.M., Lopez-Beltran A. (2018). Hypochromatic large urothelial cells in urine cytology are indicative of high grade urothelial carcinoma. APMIS.

[16] Glass R., Rosen L., Chau K., Sheikh-Fayyaz S., Farmer P., Coutsouvelis C., Slim F., Brenkert R., Das K., Raab S. (2018). Analysis of the cytomorphological features in atypical urine specimens following application of the paris system for reporting urinary cytology. Acta Cytol..

[17] Hang J.F., Charu V., Zhang M.L., VandenBussche C.J. (2017). Digital image analysis supports a nuclear-to-cytoplasmic ratio cutoff value of 0.5 for atypical urothelial cells. Cancer Cytopathol..

[18] Zhang M.L., Guo A.X., VandenBussche C.J. (2016). Morphologists overestimate the nuclear-to-cytoplasmic ratio. Cancer Cytopathol..

[19] Layfield L.J., Esebua M., Frazier S.R., Hammer R.D., Bivin W.W., Nguyen V., Ersoy I., Schmidt R.L. (2017). Accuracy and reproducibility of nuclear/cytoplasmic ratio assessments in urinary cytology specimens. Diagn. Cytopathol..

[20] Gisev N., Bell J.S., Chen T.F. (2013). Interrater agreement and interrater reliability: Key concepts, approaches, and applications. Res. Soc. Adm. Pharm..

[21] Landis J.R., Koch G.G. (1977). The measurement of observer agreement for categorical data. Biometrics.

[22] Fleiss J.L., Levin B., Paik M.C. (2003). Statistical Methods for Rates and Proportions.

[23] Sim J., Wright C.C. (2005). The kappa statistic in reliability studies: Use, interpretation, and sample size requirements. Phys. Ther..

[24] Piaton E., Advenier A.S., Carre C., Decaussin-Petrucci M., Mege-Lechevallier F., Hutin K., Nennig C., Colombel M., Ruffion A. (2017). P16/ki-67 dual labeling and urinary cytology results according to the new paris system for reporting urinary cytology: Impact of extended follow-up. Cancer Cytopathol..

[25] Virk R.K., Abro S., de Ubago J.M.M., Pambuccian S.E., Quek M.L., Wojcik E.M., Mehrotra S., Chatt G.U., Barkan G.A. (2017). The value of the UroVysion^®^ fish assay in the risk-stratification of patients with “atypical urothelial cells” in urinary cytology specimens. Diagn. Cytopathol..

